# Arthritis Among Children and Adolescents Aged <18 Years — United States, 2017–2021

**DOI:** 10.15585/mmwr.mm7229a3

**Published:** 2023-07-21

**Authors:** Tyler D. Lites, Anika L. Foster, Michael A. Boring, Elizabeth A. Fallon, Erica L. Odom, Puja Seth

**Affiliations:** ^1^Division of Population Health, National Center for Chronic Disease Prevention and Health Promotion, CDC; ^2^Oak Ridge Institute for Science and Education, Oak Ridge, Tennessee; ^3^ASRT Inc., Smyrna, Georgia.

**Summary**

**What is already known about this topic?**

Arthritis affects persons of all ages; little is known about arthritis prevalence among children and adolescents aged <18 years.

**What is added by this report?**

Approximately 220,000 children and adolescents had arthritis during 2017–2021. Prevalence increased with age and was highest among those aged 12–17 years, non-Hispanic Black or African American children and adolescents, children and adolescents with anxiety or depression, those who were physically inactive, had overweight or a heart condition, or lived in a food-insecure or smoking household.

**What are the implications for public health practice?**

Self-management interventions, physical activity or weight control, screening and linking to mental health services, and equitable access to therapies might improve arthritis outcomes in children and adolescents.

## Abstract

Arthritis affects persons of all ages, including younger adults, adolescents, and children; however, recent arthritis prevalence estimates among children and adolescents aged <18 years are not available. Previous prevalence estimates among U.S. children and adolescents aged <18 years ranged from 21 to 403 per 100,000 population depending upon the case definition used. CDC analyzed aggregated 2017–2021 National Survey of Children’s Health data to estimate the national prevalence of parent-reported arthritis diagnosed among children and adolescents aged <18 years. An estimated 220,000 (95% CI = 187,000–260,000) U.S. children and adolescents aged <18 years (305 per 100,000) had diagnosed arthritis. Arthritis prevalence among non-Hispanic Black or African American children and adolescents was twice that of non-Hispanic White children and adolescents. Co-occurring conditions, including depression, anxiety, overweight, physical inactivity, and food insecurity were associated with higher prevalences of arthritis. These findings highlight that children and adolescents should be prioritized for arthritis prevention and treatments by identifying risk factors for arthritis, developing self-management interventions to improve arthritis, physical activity or weight control, and screening and linking to mental health services. Health systems and payors can take steps to ensure equitable access to therapies (e.g., physical therapies and medications).

## Introduction

Previous estimates of the number of arthritis cases and prevalence among U.S. children and adolescents aged <18 years range from 13,400 (21 per 100,000 population) in one 1978 study using a very narrow definition of juvenile arthritis (*1*) to 294,000 (403 per 100,000) during 2001–2004 using a much broader definition of pediatric arthritis and other rheumatologic conditions (*2*). Although children and adolescents can receive diagnoses of many types of arthritis, the most common are acquired autoinflammatory diseases* that are associated with joint pain, swelling, stiffness, physical disability, and activity limitation that often persist into adulthood; depression and anxiety often co-occur with arthritis among children and adolescents (*3*,*4*,*5*). The National Survey of Children’s Health (NSCH) is an annual household survey conducted by the U.S. Census Bureau designed and funded by the Health Resources and Services Administration’s Maternal and Child Health Bureau. It is the largest national- and state-level survey of U.S. children’s health and uses a national address-based sample for online or mail collection of data from parents.^†^ NSCH asks parents about the physical and emotional health, well-being, and related factors of one randomly selected^§^^,^^¶^ child or adolescent aged <18 years from their household.

## Methods

The overall NSCH response rates during 2017, 2018, 2019, 2020, and 2021 were 37.4%, 43.1%, 42.4%, 42.4%, and 40.3%, respectively. Diagnosed arthritis was defined as parents answering “yes” to the single question, “Has a doctor or other health care provider ever told you that this child has arthritis?” These analyses included combined 2017–2021 public use deidentified data from parents who answered the question about arthritis for the selected child or adolescent, resulting in a study population of 173,406 children and adolescents aged <18 years.

Annualized, unadjusted prevalence estimates of arthritis (cases per 100,000 U.S. children and adolescents) were generated overall and by selected characteristics of the child or adolescent (e.g., demographic characteristics; depression, anxiety, or overweight; physical inactivity; and having health insurance or a place for preventive care) and characteristics of the household (e.g., parents’ highest educational attainment, whether smoking occurs in the household, and presence of food insecurity). All estimates presented were weighted to be nationally representative of the U.S. population of children and adolescents living in households.**^,^^††^ Differences in subgroups were tested against a reference group using a t-test with an a priori α-level of 0.05. Analyses accounted for the complex survey design and were conducted using SAS-callable SUDAAN (version 11.0.1; RTI International). This activity was reviewed by CDC and conducted consistent with applicable federal law and CDC policy.^§§^

## Results

During 2017–2021, an estimated 220,000 U.S. children and adolescents aged <18 years had arthritis, equating to a prevalence of 305 per 100,000 U.S. children and adolescents (Table). Arthritis prevalence increased with age, from 77 per 100,000 among children aged <6 years to 592 among those aged 12–17 years. Prevalence was higher among non-Hispanic Black or African American (Black) children and adolescents (571 per 100,000) than among non-Hispanic White (White) children and adolescents (260). Among children and adolescents with reported co-occurring conditions, prevalence was highest among those with diagnosed depression (1,980), a heart condition (1,900), or anxiety (1,310), as well as among those who had overweight (1,040). Among children and adolescents ≥6 years, arthritis prevalence was higher among those who were physically inactive (791) than those who were active 1–3 days (409), 4–6 days (282) or everyday (331). The prevalence was also higher among children and adolescents in households with food insecurity (905) or smoking (560) compared with that among children living in households without these characteristics (267 and 260, respectively). In addition, the prevalence of arthritis decreased as the level of parental educational attainment increased (534 per 100,000 among those whose parent had less than a high school education compared with 199 per 100,000 among children and adolescents with a parent with at least a 4-year college degree).

**TABLE:** Characteristics of parent-reported* diagnosed arthritis^†^ among children and adolescents aged <18 years — National Survey of Children’s Health, United States, 2017–2021

Characteristic	No. of respondents^§^	No. of children and adolescents with arthritis^§^	Weighted no. with arthritis^¶^	Prevalence** (95% CI)
**Overall (2017–2021)**	**173,406**	**568**	**220,000**	**305 (259–360)**
**Survey year**				
2017^††^	21,373	79	275,000	379 (257–558)
2018	30,132	108	193,000	267 (183–389)
2019	29,144	105	203,000	280 (199–395)
2020	42,321	136	200,000	279 (211–368)
2021	50,436	140	231,000	321 (215–478)
**Characteristic of the child or adolescent**				
**Sex**				
Male^††^	90,016	237	102,000	276 (213–359)
Female	83,390	331	118,000	335 (272–414)
**Age group, yrs**				
0–5	55,304	38	18,000	77 (31–191)^§§^
6–11	51,516	127	56,000	231 (169–317)^¶¶^
12–17^††^	66,586	403	146,000	592 (491–713)^¶¶^
Race***				
American Indian or Alaska Native	1,586	9	3,000	235 (92–598)^§§^
Black or African American	12,150	55	57,000	571 (389–839)^¶¶^
Asian or Native Hawaiian or other Pacific Islander	10,428	19	11,000	234 (99–552)^§§^
White^††^	133,494	434	125,000	260 (215–314)
Multiple races	14,364	47	22,000	339 (185–620)^§§^
Hispanic or Latino***				
No^††^	151,330	501	200,000	370 (310–443)
Yes	22,076	67	21,000	114 (79–164)^¶¶^
**Depression^†††^**				
No**^††^**	163,976	431	159,000	230 (191–277)
Yes	8,985	131	58,000	1,980 (1,380–2,830)^¶¶^
**Anxiety^§§§^**				
No**	153,924	362	138,000	209 (170–257)
Yes	18,977	202	82,000	1,310 (991–1,730)^¶¶^
**Heart condition** ^¶¶¶^				
No**^††^**	168,780	520	187,000	265 (224–313)
Yes	4,399	46	31,000	1,900 (1,080–3,320)^¶¶^
Overweight****				
No**^††^**	141,530	396	115,000	218 (179–265)
Yes	9,879	89	48,000	1,040 (705–1,540)^¶¶^
**Physically active, no. of days per wk^††††^**				
0**^††^**	11,457	99	40,000	791 (521–1,200)
1–3	46,158	199	81,000	409 (317–529)^¶¶^
4–6	34,534	138	36,000	282 (214–371)^¶¶^
Every day	24,581	81	34,000	331 (221–495)^¶¶^
**Has place for routine preventive care** ^§§§§^				
No	10,752	34	35,000	528 (297–939)^§§^
Yes^††^	161,556	524	178,000	273 (232–322)
**Has health insurance^¶¶¶¶^**				
No	7,034	26	16,000	343 (152–775)^§§^
Yes^††^	165,692	539	204,000	304 (257–359)
**Characteristic of parent or household**				
Parents’ highest educational level attained*****				
Less than high school	4,369	25	36,000	534 (296–962)^††^
High school (including GED, vocational, trade, or business school)	22,550	112	54,000	390 (268–565)
Some college or associate degree	39,150	152	58,000	378 (282–507)
4-yr college degree or higher^††^	107,337	279	72,000	199 (166–239)^¶¶^
**Household food insecurity^†††††^**				
No^††^	163,891	495	178,000	267 (225–318)
Yes	5,611	57	33,000	905 (530–1,540)^¶¶^
**Home owned** ^†††††^				
No	28,496	122	54,000	332 (240–459)
Yes^††^	123,537	367	111,000	269 (217–333)
**Smoking in household^¶¶¶¶¶^**				
No^††^	146,963	447	157,000	260 (218–310)
Yes	22,986	106	56,000	560 (370–846)^¶¶^

**Abbreviation: **GED = general educational development certificate.

* Respondent relationship to the child was defined as biologic or adoptive parent, stepparent, grandparent, foster parent, other relative, other nonrelative, or missing response.

^†^ Diagnosed arthritis was defined by answering “yes” to the question “Has a doctor or other health care provider ever told you that this child has arthritis?” The analyses excluded respondents who did not respond to the question: 216 (1.05%) during 2017, 398 (1.32%) during 2018, 289 (0.98%) during 2019, 456 (1.07%) during 2020, and 456 (0.90%) during 2021.

^§^ Categories might not sum to the respondent total because of missing responses for some characteristics.

^¶^ Weighted estimates generalize to state and national resident populations. https://www.census.gov/content/dam/Census/programs-surveys/nsch/tech-documentation/methodology/2017-NSCH-Methodology-Report.pdf; https://www2.census.gov/programs-surveys/nsch/technical-documentation/methodology/2018-NSCH-Methodology-Report.pdf; https://www2.census.gov/programs-surveys/nsch/technical-documentation/methodology/2019-NSCH-Methodology-Report.pdf; https://www2.census.gov/programs-surveys/nsch/technical-documentation/methodology/2020-NSCH-Methodology-Report.pdf; and https://www2.census.gov/programs-surveys/nsch/technical-documentation/methodology/2021-NSCH-Methodology-Report.pdf

** Cases per 100,000 U.S. children and adolescents aged <18 years.

^††^ Referent group for subgroup comparisons of arthritis prevalence.

^§§^ Estimate might be unreliable. The absolute CI width is >20%, or the relative CI width is >120% (1.2 times the estimate).

^¶¶^ T-tests were used to determine statistically significant differences in arthritis prevalence for subgroups defined by selected characteristics; differences with p≤0.05 were considered statistically significant.

*** Race was recoded from responses to the question, “What is this child’s race?” and included American Indian or Alaska Native, Black or African American, Asian or Native Hawaiian or other Pacific Islander, White, and two or more races. The 2017 and 2018 surveys included a response for “Some other race only,” which are coded as missing. Persons of Hispanic or Latino (Hispanic) origin might be of any race but are categorized as Hispanic; all racial groups are non-Hispanic. Asian and Native Hawaiian or other Pacific Islander were combined to make one racial group.

^†††^ Depression was defined by answering “yes” to the question, “Has a doctor or other health care provider ever told you that this child has depression?”

**^§§§^** Anxiety was defined by answering “yes” to the question, “Has a doctor or other health care provider ever told you that this child has anxiety?”

^¶¶¶^ Heart condition was defined by answering “yes” to the question, “Has a doctor or other health care provider ever told you that this child has a heart condition?”

**** Overweight was defined by answering yes to the question, “Has a doctor or other health care provider ever told you that this child is overweight?” The 2017 survey did not include this question.

^††††^ Physical activity was not asked of children aged ≤5 years old, and was determined by the question, “During the past week, on how many days did this child exercise, play a sport, or participate in physical activity for at least 60 minutes?”

^§§§§^ Place for routine preventive care was defined by answering “yes” to the question, “Is there a place this child usually goes when he or she needs routine preventive care, such as physical examination or well-child checkups?” Starting in 2020, the survey changed pronoun language from “he or she” to “they.”

**^¶¶¶¶^** Health insurance was determined by answering “yes” to the question, “Is this child currently covered by any kind of health insurance or health coverage plan?”

***** Parents’ highest level of education attained: less than high school (i.e., 8th grade or less or 9th–12th grade, no diploma); high school (e.g., high school graduate or GED obtained or completed a vocational, trade, or business school program); some college or associate degree (e.g., some college credit, but no degree or associate degree [Associate of Arts or Associate of Science]); 4-year college degree or higher (e.g., bachelor’s degree [Bachelor of Arts or Bachelor of Science]; master’s degree [Master of Art, Master of Science, Master of Social Work, or Master of Business Administration]; doctorate [Doctor of Philosophy or Doctor of Education]; professional degree [Doctor of Medicine, Doctor of Dental Surgery, Doctor of Veterinary Medicine, or Juris Doctorate]).

^†††††^ Household food insecurity was defined by answering “Sometimes we could not afford enough to eat” or “often we could not afford enough to eat” to the question “Which of these statements best describes your household’s ability to afford the food you need during the past 12 months?”

^§§§§§^ Home owned was defined by answering “yes” to either, “Is this house, apartment, or mobile home owned by you or someone in this household with a mortgage or loan?” or “Is this house, apartment, or mobile home owned by you or someone in this household free and clear (without a mortgage or loan)?” The 2017 survey did not collect data on homeownership.

**^¶¶¶¶¶^** Smoking in household was defined by answering “yes” to the question, “Does anyone living in your household use cigarettes, cigars, or pipe tobacco?” or “Does anyone smoke inside your home?”

## Discussion

This report found that during 2017–2021, an estimated 220,000 U.S. children and adolescents had an arthritis diagnosis, and prevalence was highest among those aged 12–17 years. Previous U.S. population estimates of arthritis among children and adolescents ranged from 13,400 to 294,000 cases, and prevalences of 21 to 403 per 100,000 population (*1*,*2*). The wide range of estimates in these studies is likely attributable to a combination of factors including the relative rarity of arthritis among children and adolescents, advances in early detection and differential diagnosis of arthritis, differences in terminology and arthritis case definitions, and variations in data sources, sampling, collection, and weighting methodologies (*1*,*2*,*5*). Whereas the current study used parent-reported health care provider diagnosis of arthritis as the case definition, previous studies (*1*,*2*) have used medical billing codes to ascertain arthritis and rheumatologic conditions among children and adolescents.

Although arthritis can affect children and adolescents of all races and ethnicities, this study identified racial and ethnic disparities. Arthritis prevalence among Black children and adolescents was twice that among those who were White. Further, prevalence of arthritis was inversely related to the highest level of parental education attained. These disparities highlight the importance of addressing social determinants of health because the impacts on health and well-being can be seen as early as childhood.

Similar to other studies, the results of this analysis determined that arthritis prevalence was high among children and adolescents with anxiety and depression. A 2019 systematic review of depression and anxiety in patients with juvenile idiopathic arthritis (*4*) found higher prevalences of symptoms of depression and anxiety among juvenile idiopathic arthritis patients and their family members than among children and adolescents without juvenile idiopathic arthritis. This review also identified a need for further data on the effect of treatment of mental health symptoms on disease outcomes among children and adolescents with juvenile idiopathic arthritis. Further, the systematic review found that children and adolescents with arthritis who were experiencing anxiety and depression also had a poorer quality of life, underscoring the need to address mental health among children and adolescents with arthritis and their families (*4*). The U.S. Preventive Services Task Force recommends screening all persons aged 8–18 years for anxiety,^¶¶^ and those aged 12–18 years for major depressive disorder.*** The rationale for routine screening is to identify youths without an anxiety diagnosis who might benefit from effective treatment for anxiety disorders.

The current study also found associations between arthritis and food insecurity as well as overweight and physical inactivity. Children and adolescents with special health care needs who are also experiencing food insecurity have been found to have increased prevalences of various negative health outcomes, including overweight or obesity (*6*). A healthy, age-appropriate diet is strongly recommended as a treatment strategy for children and adolescents with arthritis (*7*). However, more research on physical activity and weight management interventions for children and adolescents with arthritis is needed.

Arthritis therapy guidelines for children and adolescents include pharmacologic and nonpharmacologic interventions and treatments (*7*–*9*). Pharmacologic treatments include antirheumatic drugs, which help preserve joints by blocking or slowing inflammation, and nonsteroidal antiinflammatory drugs to treat stiffness, pain, and fever (*8*,*9*). The 2021 American College of Rheumatology guideline for the treatment of juvenile idiopathic arthritis recommends nonpharmacologic interventions including physical and occupational therapy to improve range of motion, muscle strength, endurance, functional deficits, and activities of daily living (*7*). Although this American College of Rheumatology guideline does not make specific physical activity recommendations, the 2018 Physical Activity Guidelines for Americans^†††^ recommend that, for optimal health and fitness, children and adolescents aged 6–17 years should engage in 60 minutes of daily moderate-to-vigorous physical activity. Physically active children and adolescents experience improved cognition and fitness, stronger bones and muscles, have lower percentages of body fat, and lower risk for depression compared with inactive children and adolescents. Although preventing some types of arthritis among children and adolescents is challenging, early diagnosis and prompt treatment might prevent permanent joint damage, improve health outcomes, reduce health disparities, and maintain quality of life (*10*).

### Limitations

The findings in this report are subject to at least five limitations. First, because of the cross-sectional nature of this survey, causality among selected characteristics and arthritis prevalence cannot be inferred. Second, parent-reported arthritis diagnoses cannot be validated by medical records. Third, recall and social desirability biases or lack of knowledge about arthritis or other health conditions might result in misclassification. Fourth, because of the rarity of arthritis among children and adolescents, estimates for all subgroups might not be stable or precise, as evidenced by the wide CIs. Finally, the single survey question about an arthritis diagnosis does not provide the opportunity to estimate the prevalence of or distinguish among arthritis subtypes and does not assess undiagnosed arthritis cases.

### Implications for Public Health Practice

This study combined data across 5 years resulting in a large sample size, providing stable prevalence estimates of arthritis among U.S. children and adolescents with the most recently available data and filling a gap in nationally representative, population-based estimates of arthritis among children and adolescents. The findings from this report highlight children and adolescents to prioritize for arthritis prevention and treatment by identifying risk factors for arthritis among children and adolescents, developing self-management interventions to improve childhood arthritis, physical activity or weight control, and screening and linking children and adolescents to needed mental health services. Addressing social determinants of health and systemic factors that might contribute to disparities in arthritis prevalence needs to be prioritized. Health systems and payors can take steps to ensure equitable access to therapies (e.g., physical therapies and medications).

## Footnotes

* The seven most common autoinflammatory arthritis types among children and adolescents include 1) oligoarticular juvenile idiopathic arthritis, 2) polyarticular juvenile idiopathic arthritis–rheumatoid factor negative, 3) polyarticular juvenile idiopathic arthritis–rheumatoid factor positive, 4) enthesitis-related juvenile idiopathic arthritis, 5) psoriatic juvenile idiopathic arthritis, 6) systemic juvenile idiopathic arthritis, and 7) undifferentiated arthritis.

† Respondent relationship to the child was defined as biologic or adoptive parent, stepparent, grandparent, foster parent, other relative, other nonrelative, or missing response.

§
https://www.census.gov/programs-surveys/nsch.html

¶
https://mchb.hrsa.gov/data-research/national-survey-childrens-health

** The National Survey of Children’s Health is weighted to be representative of the U.S. population of noninstitutionalized persons aged ≤17 years. https://www.census.gov/content/dam/Census/programs-surveys/nsch/tech-documentation/methodology/2017-NSCH-Methodology-Report.pdf; https://www2.census.gov/programs-surveys,nsch/technical-documentation/methodology/2018-NSCH-Methodology-Report.pdf; https://www2.census.gov/programs-surveys/nsch/technical-documentation/methodology/2019-NSCH-Methodology-Report.pdf; https://www2.census.gov/programs-surveys/nsch/technical-documentation/methodology/2020-NSCH-Methodology-Report.pdf; and https://www2.census.gov/programs-surveys/nsch/technical-documentation/methodology/2021-NSCH-Methodology-Report.pdf

††
https://www2.census.gov/programs-surveys/nsch/technical-documentation/methodology/NSCH-Guide-to-Multi-Year-Estimates.pdf

§§ 45 C.F.R. part 46.102(1)(2), 21 C.F.R. part 56; 42 U.S.C. Sect. 241(d); 5 U.S.C. Sect. 552a; 44 U.S.C. Sect. 3501 et seq.

¶¶
https://www.uspreventiveservicestaskforce.org/uspstf/recommendation/screening-anxiety-children-adolescents

***
https://www.uspreventiveservicestaskforce.org/uspstf/recommendation/screening-depression-suicide-risk-children-adolescents

†††
https://health.gov/sites/default/files/2019-09/Physical_Activity_Guidelines_2nd_edition.pdf

## References

[1] Gewanter HL, Roghmann KJ, Baum J. The prevalence of juvenile arthritis. Arthritis Rheum 1983;26:599–603. 10.1002/art.1780260504 6847723

[2] Sacks JJ, Helmick CG, Luo YH, Ilowite NT, Bowyer S. Prevalence of and annual ambulatory health care visits for pediatric arthritis and other rheumatologic conditions in the United States in 2001–2004. Arthritis Rheum 2007;57:1439–45. 10.1002/art.23087 18050185

[3] Simon TA, Harikrishnan GP, Kawabata H, Singhal S, Brunner HI, Lovell DJ. Prevalence of co-existing autoimmune disease in juvenile idiopathic arthritis: a cross-sectional study. Pediatr Rheumatol Online J 2020;18:43. 10.1186/s12969-020-00426-9 32503658 PMC7275412

[4] Fair DC, Rodriguez M, Knight AM, Rubinstein TB. Depression and anxiety in patients with juvenile idiopathic arthritis: current insights and impact on quality of life, a systematic review. Open Access Rheumatol 2019;11:237–52. 10.2147/OARRR.S174408 31807093 PMC6830373

[5] Helmick CG, Felson DT, Lawrence RC, ; National Arthritis Data Workgroup. Estimates of the prevalence of arthritis and other rheumatic conditions in the United States: part I. Arthritis Rheum 2008;58:15–25. 10.1002/art.23177 18163481

[6] Khanijahani A, Pawcio S. Household food insecurity and childhood obesity/overweight among children with special healthcare needs: results from a nationally representative sample of 10–17 years old U.S. children. Pediatr Obes 2023;18:e13015. 10.1111/ijpo.13015 36825692

[7] Onel KB, Horton DB, Lovell DJ, 2021 American College of Rheumatology guideline for the treatment of juvenile idiopathic arthritis: recommendations for nonpharmacologic therapies, medication monitoring, immunizations, and imaging. Arthritis Care Res (Hoboken) 2022;74:505–20. 10.1002/acr.24839 35233989 PMC10231687

[8] Ringold S, Angeles-Han ST, Beukelman T, 2019 American College of Rheumatology/Arthritis Foundation guideline for the treatment of juvenile idiopathic arthritis: therapeutic approaches for non-systemic polyarthritis, sacroiliitis, and enthesitis. Arthritis Rheumatol 2019;71:846–63. 10.1002/art.40884 31021537 PMC6561114

[9] Onel KB, Horton DB, Lovell DJ, 2021 American College of Rheumatology guideline for the treatment of juvenile idiopathic arthritis: therapeutic approaches for oligoarthritis, temporomandibular joint arthritis, and systemic juvenile idiopathic arthritis. Arthritis Care Res (Hoboken) 2022;74:521–37. 10.1002/acr.24853 35233986 PMC10124899

[10] Marzan KA, Shaham B. Early juvenile idiopathic arthritis. Rheum Dis Clin North Am 2012;38:355–72. 10.1016/j.rdc.2012.04.006 22819089

