# *Erratum*: Vol. 72, No. 29

**DOI:** 10.15585/mmwr.mm7317a5

**Published:** 2024-05-02

In the report, “Arthritis Among Children and Adolescents Aged <18 Years — United States, 2017–2021,” on page 790, there was an error in the Table. Under Characteristic, under Race,*** the third entry should have read, “**Asian or Native Hawaiian or other Pacific Islander**.”

The ninth table footnote should have read, “*** Race was recoded from responses to the question, “What is this child’s race?” and included American Indian or Alaska Native, Black or African American, **Asian or** Native Hawaiian or other Pacific Islander, White, and two or more races. The 2017 and 2018 surveys included a response for “Some other race only,” which are coded as missing. Persons of Hispanic or Latino (Hispanic) origin might be of any race but are categorized as Hispanic; all racial groups are non-Hispanic. **Asian and Native Hawaiian or other Pacific Islander were combined to make one racial group**.”

